# Burden and determinants of anemia among lactating women in Ethiopia: evidence from demographic health survey

**DOI:** 10.1038/s41598-024-65583-3

**Published:** 2024-06-27

**Authors:** Selamawit Girma, Neil Abdureshid, Ketema Ayele, Imam Dagne, Berhanu Abebaw Mekonnen, Shambel Abate, Aragaw Hamza, Milkias Solomon, Abdu Oumer

**Affiliations:** 1 Department of Public Health, College of Medicine and Health Sciences, Dire Dawa University, Dire Dawa, Ethiopia; 2 Department of Midwifery, College of Medicine and Health Sciences, Dire Dawa University, Dire Dawa, Ethiopia; 3 Department of Nutrition and Dietetics, School of Public Health, Bahir Dar University, Bahir Dar, Ethiopia; 4 Department of Anesthesia, College of Medicine and Health Sciences, Dire Dawa University, Dire Dawa, Ethiopia

**Keywords:** Anemia, Demographic health survey, Determinants, Ethiopia, Lactating women, Nutrition, Public health

## Abstract

Globally one-third of global population are victims of anemia, significantly impacting maternal and infant health and linked to poor cognition, productivity, and mortality risks. We used randomly selected 4040 lactating mothers’ record from nationally representative survey. Descriptive statistics were weighted, and the standard hemoglobin cutoff point (below 12 g/dl) was used. Bivariable and multivariable multilevel binary logistic regression model considering the individual and community-level factors associated with anemia was employed. Crude and adjusted odds ratios with a 95% confidence interval were reported. In Ethiopia, 32.3% (95% CI 30.9–33.7%) of lactating women were anemic, with 23.4% having mild, 7.3% moderate, and 1.2% severe anemia. Pastoral regions (Afar, Somalia, and Oromia region) had higher burden of anemia than the others. The advanced age of the mother above 45 years (AOR = 1.43 (1.11–1.82), unemployment (AOR = 1.19; 95% CI 1.08–1.32), household wealth index (AOR = 0.56; 95% CI 0.50–0.63), extended family size (AOR = 1.20; 95% CI 1.04–1.46), and not using family planning (AOR = 1.70; 95% CI 1.49–1.93) were significant factors associated with anemia. Anemia is a moderate public health problem and associated with location and other factors to be addressed via effective interventions.

## Introduction

Lactation is one of the most nutrient demanding stages of human development and it is a critical stage, where blood loss and increased nutrient demand predispose them to maternal depletion and increased risk of anemia. Anemia is a reduced hemoglobin level in the blood below the World Health Organization (WHO) cutoff of 12 g/dl for non-pregnant women in reproductive age groups, including lactating women^1^. Anemia is affects one-third of the global population^2–4^ where the causes are multiple and nutritional causes play a great role. Among these, iron deficiency is the single most important contributor to anemia, contributing to more than 40%^5^.

Furthermore, anemia is a moderate to severe public health problem in more than 142 countries. An estimated 529 million (29.4%) of women of reproductive age are victims of anemia due to blood loss during menstruation, pregnancy, childbirth and other factors^6^. Globally, about 40% of children under five years of age, 37% among pregnant women, and 30% of women of reproductive age are affected by anemia^7^. In Ethiopia, iron deficiency affects about 8% of the population with wide variations among regions (6–48%). Moreover, 13% of women of reproductive age had anemia where the regional variations range from 6 to 40%, making mild to moderate public health problem^8^.

Anemia is strongly linked to negative growth consequences such as stunting, reduced cognition, morbidity, mortality, and significant economic loss^9^. Anemia is a huge concern globally, contributing to more than 1 million annual deaths, mainly from Africa and Southeast Asia (70%)^3,7,10^. Aside from the obvious link to pregnancy complications, it has also been linked to depression, stress, and cognitive functioning in lactating women^3^.

On the other side, lactation is a period where women are highly susceptible to iron depletion in the presence of an inadequate dietary supply^11,12^. The maternal iron store is greatly depleted by repeated reproductive burden, blood loss during pregnancy, and childbirth^11,13^. In addition, the subsequent iron loss through breast milk at the expense of maternal iron store further aggravates the problem^9^. These problems are more prevalent in Africa, where the fertility rate is higher and dietary intake is poor^2,3,14^. These is strongly associated with the prevailing occurrence of anemia among this segment of the population that is being further aggravated by multiple dynamic changes happening across the globe.

Ethiopia faces multiple environmental, economic, health and dietary changes influenced by multiple dimensions that could affect nutritional status, anemia specifically^15,16^. Maternal and child nutrition vulnerability related to anemia arises from widespread iron deficiency anemia, inadequate dietary diversity, and limited healthcare access^9^. Traditional diets centered around staples and monotonous diets could limit iron intake and bioavailability. Household food security vulnerability is evident during seasonal shortages, affecting many families^3^. With all these vulnerabilities could further aggravate the existing burden of anemia in Ethiopia^17,18^.

Much of previous efforts were among pregnant women with a prevalence estimate of above 50%^13,16,18–20^ and yet limited evidence exist among lactating women in Ethiopia. The previous consecutive national reports indicated that 29.9% (2005) and 18.5% (2011) of lactating women had anemia in Ethiopia^21^. However, lactation is immediate postpartum period where the problem of pregnancy usually continues and is associated with poor maternal and child health^2,9^. Anemia during this period is of great concern from maternal and newborn perspectives^16,22,23^. It is linked to poor infant growth^24^, depression, and adverse maternal and child outcomes^25^. Comprehensive evidence on anemia could contribute to the sustainable development goals by reducing morbidity and mortality. of women during the lactation period associated with anemia^17^.

For a better understanding of the contributing factors to anemia among lactating women, a robust analysis of existing large nationally representative datasets is very informative and conclusive as compared to local pocket studies. The DHS gives free access to the country specific survey datasets, enables them to characterize and assess the potential interregional variations and important modifiable risk factors for the prevention of anemia. This paper was to determine the burden, regional distribution, and risk factors of anemia among lactating women in Ethiopia.

## Methods

### Data sources

The current paper is based on secondary analysis of the 2016 EDHS survey, spanning all regional states and the two federal administrative cities of Ethiopia. Ethiopia is a country that is located in the eastern parts of Africa, within Sub-Saharan Africa. An estimated total population of more than 110 million people resides in the country. The survey was conducted in 2016 from January to June. The data were collected from a nationally representative sample of households in a way that allows national and regional levels estimates of indicators. The results of this analysis targeted all lactating mothers (currently breast-feeding mothers during the data collection period) based on a representative sample of lactating mothers from a randomly selected household. The data nature and coding were reviewed thoroughly to extract important variables and observations. Considering the breast-feeding status during the interview (V404) and the pregnancy status, we extracted 4252 data of lactating women interviewed for the survey. The missing values for the outcome variable was checked, and all cases of the lactating women were included in the analysis (n = 4040) Data was captured from a representative enumeration area and 11 administrative regions of the country.

### Variables of the study

The anemia status of the lactating mother was a dependent variable while many socio-demographic, obstetric, diet-related, and other related variables were considered as potential independent variables in the study. Some of the variables included were region, residence, age of the mother, household wealth index, maternal occupation, mother’s educational status, husband’s education, family planning use, marital status, nutritional status of the mother, substance use (alcohol, cigarette and khat chewing).

It is known that different cut-off points are used to diagnose anemia based on the WHO standard anemia (based on hemoglobin measurement) classification adjusted for different life stages (pregnancy, childhood, women of reproductive age and adult males), smoking status, and altitude. Hence, for lactating women who are within women of reproductive age group, hgb concentration value below 12.0 g/dl were classified as anemic. Moreover those with 10–11.9 g/dl as mild, 7–9.9 g/dl as moderately anemic and below 7 g/dl as severely anemic^1^ (Table 1).

**Table 1:** Summary of the study variables and measurements.

Variables	Measurements
Outcome variable	
Anemia	Anemia was measured based on the Hgb level measured in standardized way. Those lactating mothers with Hgb below 12 g/dl were classified as anemic and non-anemic otherwise
Independent variables	
Region, residence, maternal occupation, mother’s education, husband’s education, family planning use, household family size, marital status, age, substance use (alcohol, cigarette, and khat chewing)attendance	These data were captured using interviewer administered questionnaire and through face-to- face interview
Household wealth index	Collected using the extended household asset questionnaire and the wealth index was constructed using the principal component analysis
Anthropometric measurements (Body mass index)	The weight and height of mothers was captured using standardized anthropometric measurement and used to assess nutritional status based on body mass index, where below 18.5 (undernutrition), 18.5–24.9 (normal), 25–29.9 (overweight) and above ≥ 30 (obese)

### Sampling procedures

A two-stage stratified sampling was employed to include households from a total of 84,915 enumeration areas (EAs) prepared from the 2007 census. An EA is a geographic area covering an average of 181 households per day. Samples were taken from the nine regions and administrative regions of the country. Each primary sampling unit was stratified into urban and rural areas. Then, samples of EAs were selected from the stratum in two stages. Proportional to size sample allocation techniques were maintained at region, zone, and district, where the respective number of enumeration areas were sampled accordingly.

### Data collection methods

Data was collected through face-to-face interview and measurements via paper-based and/or tablets. Pretested tool with a translation into local languages was used. Women’s questionnaire that measures socio-demographic characteristics of the mothers, reproductive health, and service use behaviors were included. In addition, biomarkers: anthropometry (body mass index), and anemia testing (hemoglobin) were included. Hemoglobin measurement was done using battery-operated portable HemoCue analyzer for all anemia samples. A 5 ml blood sample was taken after obtaining a written consent from the study subjects^26^.

A total of 132 individuals were selected as interviewers, 66 as biomarker technicians, 33 as field editors, and 33 as team supervisors, with a four-day field practice being organized. Instruction on how to administer the paper and electronic questionnaires, mock interviews between participants in the classroom, and practice interviews with real respondents in areas outside the survey sample were conducted. In addition, laboratory professionals collected the biomarker data under standard procedures^26^.

### Data analysis

The data was acquired in SPSS format and the analysis was done in SPSS version 20. The data was presented in frequency, percentage, tables, and graphs. The body mass index (kg m^−2^) was calculated and classified as underweight (less than 18.5 kg m^−2^), normal (18.5–24.9 kg m^−2^), and overweight (above 25 kg m^−2^). The household asset variables were analyzed using a principal component analysis after checking that all assumptions were carried out. Then, the factor score is used to categorize individuals into wealth quintiles (poorest, poorer, middle, rich, and richest). For the present analysis, the wealth index was presented into three groups (poor, middle, and rich) better presentation.

A bivariable and multivariable multilevel binary logistic regression analysis was carried out to identify determinants of anemia. Variables found statistically significant at p-value below 0.25 during bivariable analysis were analyzed in the multivariable logistic regression model. Crude and an adjusted odds ratio with a 95% confidence level was calculated. Statistical significance was declared at a p-value below 0.05. Multicollinearity and effect modifications were evaluated accordingly using variance inflation factor (above 10) and interaction effects.

### Ethical approval and consent to participate

The dataset was obtained from the DHS site with a full legal request. The dataset will not be shared with the third party. These are anonymous surveys, which do not allow any potential identification of any single household or individual in the data file. The survey protocol, including biomarker sample collection, was reviewed and approved by the Federal Democratic Republic of Ethiopia's Ministry of Science and Technology and the Institutional Review Board of ICF International. Written informed consent was obtained from all subjects and/or their legal guardian for those aged less than 18 years. The accessed data were used for the purpose of registered research paper. Confidentiality of the data was kept and no effort made to identify any household or individual respondent interviewed in the survey. The data were not passed on to other researchers without the written consent of DHS. The data were fully accessed at www.dhsprogram.com. All methods were carried out in accordance with Declaration of Helsinki.

## Results

### Socio-demographic and economic characteristics

**Figure 1:**
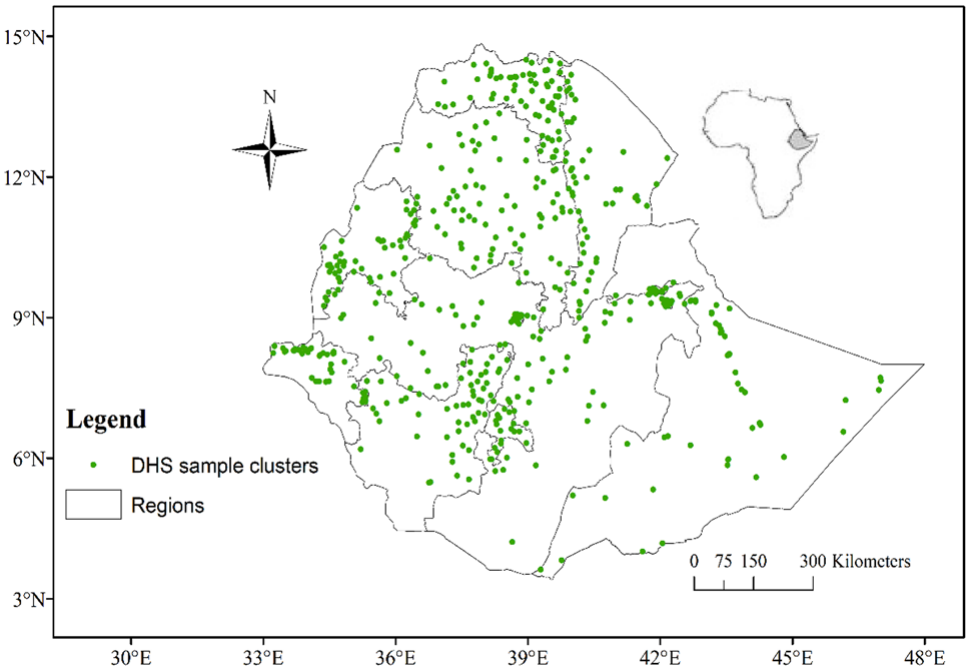
Sample cluster (enumeration areas) distribution for sample household for the 2016 EDHS in Ethiopia.

Out of the total 4252 records, 4040 (95% retrieval rate) records of lactating women were included in the analysis after excluding 67 (1.6%) records due to anemia status not available although sample were collected and 145 (3.4%) were excluded due to maternal refusal or to give blood sample at the start. A total of 800 (18.8%) were from urban areas, and the majority (47.1%) were aged 26–35 years of age. Around 2431 (63.1%) had no education at all (illiterate), and 1683 (43.6%) were Muslims. Almost one-third, or 1439 (33.8%) of lactating women were living in the poorest wealth quintile, while only 559 (13.1%) were from the richest quintile. The majority, 3994 (94%) and 2331 (57.7%), were married and unemployed, respectively. About 648 (15.2%), (13.4%), and (12.3%) were from the Oromia, southern, and Amhara regions of Ethiopia (Fig. 1).

### Burden and distribution of *anemia*

**Figure 2:**
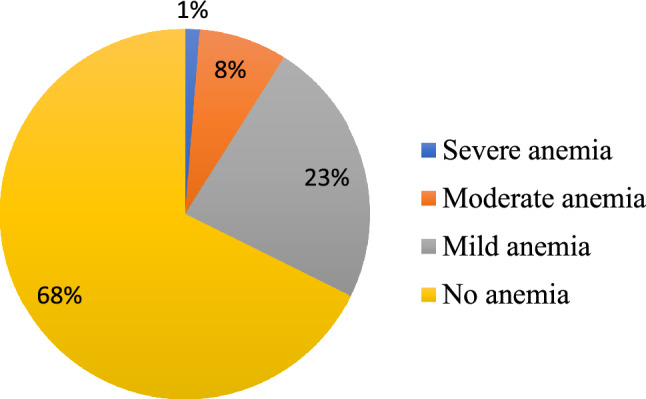
Prevalence and levels of anemia among lactating women in Ethiopia, based on the 2016 national DHS data for Ethiopia.

In Ethiopia, 1306 (32.3%; 95% CI 30.9–337%) of lactating women were anemic; 23.4%, 7.3%, and 1.2% had mild, moderate, and severe anemia, respectively. A wide interregional variation in the prevalence of anemia was observed among regions where pastoral regions (Afar, Somalia, and Parts of Oromia regions) had the highest prevalence of anemia (Fig. 2).

Anemia among lactating women was more prevalent among women residing in rural area and in the age group of 26–35 years as compared to counterparts. Women in the lowest socioeconomic class were the most prone to anemia. In addition, the burden of anemia was higher among married and uneducated women (Table 2).

**Table 2:** Prevalence and degree of anemia by different sociodemographic and socioeconomic characteristics of lactating women in Ethiopia.

variables	Categories	Frequency (%)	levels of anemia			
			Severe	Moderate	Mild	No anemia
Residence	Urban	738 (78.2%)	1 (0.14%)	35 (4.74%)	125 (16.94%)	577 (78.18%)
	Rural	3302 (65.3%)	50 (1.51%)	276 (8.36%)	819 (24.8%)	2157 (65.32%)
Age of women	15–25	1493 (67.8%)	20 (1.34%)	127 (8.51%)	333 (22.3%)	1013 (67.85%)
	26–35	1915 (66.6%)	25 (1.31%)	153 (7.99%)	462 (24.13%)	1275 (66.58%)
	36–45	606 (70.8%)	6 (0.99%)	29 (4.79%)	142 (23.43%)	429 (70.79%)
	≥ 46	26 (65.4%)	0 (0%)	2 (7.69%)	7 (26.92%)	17 (65.38%)
Household wealth index	Poor	2069 (59.7%)	43 (2.08%)	222 (10.73%)	569 (27.5%)	1235 (59.69%)
	Middle	617 (72.1%)	2 (0.32%)	29 (4.7%)	141 (22.85%)	445 (72.12%)
	Rich	1444 (79.2%)	6 (0.42%)	60 (4.16%)	234 (16.2%)	1144 (79.22%)
Educational level of women	Illiterate	2431 (63.5%)	47 (1.93%)	227 (9.34%)	613 (25.22%)	1544 (63.51%)
	Primary education	1150 (73%)	3 (0.26%)	67 (5.83%)	241 (20.96%)	839 (72.96%)
	Secondary education	317 (76.3%)	0 (0%)	10 (3.15%)	65 (20.5%)	242 (76.34%)
	Higher education	142 (76.8%)	1 (0.7%)	7 (4.93%)	25 (17.61%)	109 (76.76%)
Marital status of women	Single	212 (71.2%)	0 (0%)	11 (5.19%)	50 (23.58%)	151 (71.23%)
	Married	3797 (67.4%)	51 (1.34%)	298 (7.85%)	889 (23.41%)	2559 (67.4%)
Occupational status of women	Yes	1709 (73%)	10 (0.59%)	84 (4.92%)	367 (21.47%)	1248 (73.03%)
	No	2331 (63.7%)	41 (1.76%)	227 (9.74%)	577 (24.75%)	1486 (63.75%)
Household family size	< 4	1256 (72.9%)	11 (0.88%)	86 (6.85%)	244 (19.43%)	915 (72.85%)
	5–8	2295 (66%)	33 (1.44%)	185 (8.06%)	562 (24.49%)	1515 (66.01%)
	> 8	489 (62.2%)	7 (1.43%)	40 (8.18%)	138 (28.22%)	304 (62.17%)

Anemia was highly prevalent in Somalia's regional states (67.2%), followed by Afar (48.6%) and Dire Dawa (43.2%). Specifically severe anemia was higher in Somalia (6.5%) and Afar (2.5%). However, mild anemia was more prevalent across all regional and city administrations. Furthermore, in certain regions like Addis Ababa and Gambelia, no cases of severe anemia were identified (Table 3).

**Table 3:** Prevalence and degree of anemia estimates disaggregated by region among lactating women in Ethiopia.

Region	Number	Anemia %	levels of anemia			
			Severe	Moderate	Mild	No anemia
Tigray	449	26.7%	1 (0.22%)	20 (4.45%)	99 (22.05%)	1 (0.22%)
Amhara	518	22.8%	1 (0.22%)	15 (3.34%)	102 (22.72%)	1 (0.22%)
Oromia	621	31.2%	9 (2%)	39 (8.69%)	146 (32.52%)	9 (2%)
SNNP	556	23.0%	2 (0.45%)	26 (5.79%)	100 (22.27%)	2 (0.45%)
Somalia	369	67.2%	24 (5.35%)	99 (22.05%)	125 (27.84%)	24 (5.35%)
Afar	315	48.6%	8 (1.78%)	46 (10.24%)	99 (22.05%)	8 (1.78%)
Gambella	292	33.6%	0 (0%)	17 (3.79%)	81 (18.04%)	0 (0%)
Benshangule	364	23.1%	1 (0.22%)	9 (2%)	74 (16.48%)	1 (0.22%)
Dire Dawa	190	43.2%	2 (0.45%)	26 (5.79%)	54 (12.03%)	2 (0.45%)
Harari	187	29.4%	3 (0.67%)	10 (2.23%)	42 (9.35%)	3 (0.67%)
Addis Ababa	179	14.5%	0 (0%)	4 (0.89%)	22 (4.9%)	0(0%)

### Factors associated with *anemia* among lactating mothers

In bivariable analysis, the age of the mother, residence, maternal education, household wealth index, household family size, marital status, and alcohol use was associated with anemia in the crude model. Women aged 26 to 35 years (COR = 1.25; 95% CI 1.03–1.52), living in rural areas (COR = 1.80; 95% CI 1.65–1.95), and being married (COR = 1.25; 95% CI 1.2–13) had a 25%, 80%, and 25% higher risk of anemia as compared to counterparts, respectively. Women in a higher the higher wealth quintile (COR = 0.42; 95% CI 0.38–0.48) and had lower risks of anemia compared to lower wealth quintile in the crude model. Women in the higher family size had a 40% increased risk of being anemic (COR = 1.40; 95% CI 1.25–1.60). Concerning substance use, alcohol consumption is associated with reduced risk of anemia (COR = 0.51; 95% CI 0.46–0.55) while association with khat chewing and cigarette smoking did not show statistical significance. In addition, the risk of anemia was found to be higher among undernourished women (COR = 1.29; 95% CI 1.16–1.34) and lower among obese women (COR = 0.66; 95% CI 0.52–0.84) compared to well-nourished mothers in the unadjusted model. Those lactating mothers who never used any family planning methods (COR = 2.36; 95% CI 2.06–2.70) and unemployed mothers (AOR = 1.54; 95% CI 1.34–1.76) had 2.36 and 1.54 times higher odds of anemia compared to counterparts (Table 4).

**Table 4:** Unadjusted logistic regression model for factors associated with anemia among lactating women in Ethiopia.

Variables	Categories	Anemia among LW		COR	P-value
		Yes (n = 1306)	No (n = 2623)		
Age	15–25	480 (32.2%)	1013 (67.8%)	1	
	26–35	660 (34.1%)	1275 (65.9%)	0.92 (1.04-.86)	0.63
	36–45	177 (29.2%)	429 (70.8%)	1.25 (1.03–1.52)	0.02**
	>=46	9 (34.6%)	17 (65.4%)	1.03 (0.92–1.13-.)	0.22
Residence	Urban	161 (21.8%)	577 (78.2%)	1	
	Rural	1145 (34.7%)	2157 (65.3%)	1.80 (1.65–1.95)	0.0001***
Educational status of the women	Illiterate	887 (36.5%)	1544 (63.5%)	1	
	Primary education	311 (27%)	839 (73%)	0.59 (0.54–0.64)	0.0001***
	Secondary education	242 (17.4%)	1150 (82.6%)	0.46 (0.29–0.41)	0.0001***
	Higher education	33 (23.2%)	109 (76.8%)	0.34 (1.09–1.64)	0.0001**
Household wealth index	Poor	834 (40.1%)	1245 (59.9%)	1	
	Middle	172 (27.9%)	445 (72.1%)	0.58 (0.58–0.52)	0.001***
	Rich	300 (22.3%)	1044 (77.7%)	0.42 (0.38–0.48)	0.001***
Marital status	Single	68 (28%)	175 (72%)	1	
	Married	1238 (32.6%)	2559 (67.4%)	1.25 (1.2–13)	0.0001***
Occupation of the women	Employed	461 (27%)	1248 (73%)	1	
	Unemployed	845 (36.3%)	1486 (63.7%)	1.54 (1.34–1.76)	0.0001***
Household family size	<=4	341 (27.1%)	915 (72.9%)	1	
	5–8	780 (34%)	1515 (66%)	1.30 (1.23–1.45)	0.0001***
	>=8	185 (37.8%)	304 (62.2%)	1.40 (1.25–1.60)	0.0001***
Husband education	Illiterate	669 (37.5%)	1116 (62.5%)	1	
	Primary	394 (29%)	964 (71%)	0.66 (0.60–0.73)	0.0001***
	Secondary	115 (28.5%)	289 (71.5%)	0.63 (0.55–0.73)	0.0001***
	Higher	76 (25.9%)	218 (74.1%)	0.52 (0.44–0.61)	0.0001***
Cigarette smoking	No	1295 (32.3%)	2710 (67.7%)	1	
	Yes	11 (31.4%)	24 (68.6%)	0.96 (0.75–1.11)	0.590
Khat chewing	No	1174 (32.1%)	2483 (67.9%)	1	
	Yes	132 (34.5%)	251 (65.5%)	1.11 (0.87–1.23)	
Alcohol drinking	No	1010 (36.5%)	1757 (63.5%)	1	
	Yes	296 (23.3%)	977 (76.7%)	0.51 (0.46–0.55)	0.0001***
BMI category	Low	476 (36.6%)	824 (63.4%)	1.29 (1.16–1.34)	0.001***
	Normal	744 (30.9%)	1664 (69.1%)	1	
	Over weight	65 (25%)	195 (75%)	0.60 (0.52–0.70)	0.001***
	Obese	21 (29.2%)	51 (70.8%)	0.66 (0.52–0.84)	0.01**
FP use	Ever used	459 (23%)	1533 (77%)	1	
	Never used	847 (41.4%)	1201 (58.6%)	2.36 (2.06–2.70)	0.0001**

p-value below 0.05 (*), 0.005 (**), and 0.0005 (***). BMI was categorized as low or undernourished (BMI below 18.5 kg/m^2^), normal (18.5–24.9 kg/m^2^), overweight (25–29.9 kg/m^2^) and obese (> 30 kg/m^2^).

After adjusting for potential confounders, a stepwise multivariable logistic regression model was fitted (Hosmer–Lemeshow’s p-value = 0.89). The age of the mother, household wealth index, educational status of the women, occupation of the women, household family size, and family planning use were significantly associated with anemia. Women with higher wealth quintile (AOR = 0.56; 95% CI 0.50–0.63) had a lower odd of anemia than women with lower socioeconomic status. Better husband education showed to proportionally decrease the odds of anemia among lactating women as compared to those without formal education (P-value < 0.0001) where those who attended primary (AOR = 0.79; 95% CI 0.70–0.90), secondary (AOR = 0.82; 95% CI 0.72–1.10) and higher education secondary (AOR = 0.75; 95% CI 0.56–1.03) had 21%, 18% and 25% reduced odds of anemia, respectively. Unemployed mothers (AOR = 1.19; 95% CI 1.08–1.32) and women with extended family size (household size) above seven (AOR = 1.20; 95% CI 1.04–1.46) had about 20% higher risks of anemia compared to employed and mothers with smaller household size. Those who did not use family planning (AOR = 1.7; 95% CI 1.49–1.93) was associated with increased risks of anemia among lactating mother (Table 5).

**Table 5:** Multivariable logistic regression output for adjusted factors associated with anemia among lactating women in Ethiopia.

Variables	Categories	Anemia		AOR	P-value
		Yes	No		
Age in years	15–25	480 (32.2%)	1013 (67.8%)	1	1
	26–35	660 (34.1%)	1275 (65.9%)	1.09 (0.94–1.19)	0.948
	36–45	177 (29.2%)	429 (70.8%)	1.25 (1.08–1.43)	0.004**
	>=46	9 (34.6%)	17 (65.4%)	1.43 (1.11–1.82)	0.006**
Educational status of the mother	Illiterate	887 (36.5%)	1544 (63.5%)	1	1
	Primary education	311 (27%)	839 (73%)	0.79 (0.70–0.90)	0.0001***
	Secondary education	242 (17.4%)	1150 (82.6%)	0.82 (0.72–1.10)	0.308
	Higher education	33 (23.2%)	109 (76.8%)	0.75 (0.56–1.03)	0.086
Wealth index	Poor	834 (40.1%)	1245 (59.9%)	1	1
	Middle	172 (27.9%)	445 (72.1%)	0.7 (0.60–0.80)	0.0001***
	Rich	300 (22.3%)	1044 (77.7%)	0.56 (0.50–0.63)	0.0001***
Occupation of the mother	Employed	461 (27%)	1248 (73%)	1	1
	Unemployed	845 (36.3%)	1486 (63.7%)	1.19 (1.08–1.32)	0.001***
House hold size	<=4	341 (27.1%)	915 (72.9%)	1	1
	5–8	780 (34%)	1515 (66%)	1.20 (1.06–1.32)	0.003**
	>=8	185 (37.8%)	304 (62.2%)	1.20 (1.04–1.46)	0.014*
FP use	ever used	459(23%)	1533 (77%)	1	1
	never used	847 (41.4%)	1201 (58.6%)	1.70 (1.49–1.93)	0.0001***

p-value below 0.05 (*), 0.005 (**), and 0.0005 (***).

## Discussions

The findings of this study showed that 32.3% of lactating women had anemia indicating anemia a moderate public health problem in Ethiopia. Also, the advanced age of the mother above 45 years unemployment, household wealth index, extended family, and not using family planning were significant factors associated with anemia.

The current estimate is slightly higher than the 2005 (29.9%) and 2011 national DHS (18.5%)^21^. However, a relatively consistent result was found as compared to the study done in Madrid (29%)^27^. In contrast, studies conducted in India (60–82%)^24^, Myanmar (60.3%)^28^, and Vietnam (39.0%)^29^, showed a higher burden of anemia among lactating women. Sub-national evidence has also showed that anemia affects 43%^13^ and 28.7% of lactating women in the Northwest and South West Ethiopia, which is above the estimate in Amhara (12.3%) and the Oromia region (15.3%)^30^. Furthermore, a great inter-regional variation in anemia prevalence was observed^12^, where anemia is more prevalent in the pastoral and agropastoral regions, which could be linked to many dietary and non-dietary factors. For instance, this might be related to a higher reliance on cow or camel milk, which is a rich source of calcium, affecting iron absorption negatively. In addition, we noted a persistently increasing burden of anemia from 2005 to 2016 in Ethiopia^21^, which indicates that the existing interventions might not be effective and due attention is not given for lactating women. On the contrary, more recent estimates depicted that 13% of women in reproductive age had anemia with a consistently higher occurrence among women from Somalia (40%) and Dire Dawa^30^.

This may be associated with differences in socio-economic status and dietary behaviors^2,11,13,22^. In addition, the current study is based on a country wide large-scale survey, which is inclusive of women from multiple socioeconomic and dietary habits contexts^12,29^. It is partly explained by the increased blood loss during delivery, coupled with poor dietary habits and prevailing maternal malnutrition further predisposing women to higher risks of anemia^9,20,22,23^. Hence, anemia during pregnancy which persists while lactation could potentially increase the risks maternal morbidity, mortality, maternal cognition, and poor childhood development, which warrants context-specific interventions addressing anemia like tailored iron and folic acid supplementation schedules Overall, this study indicated that anemia among lactating women is an important public health problem, that needs to be addressed through targeted interventions^7,15^. Larger family size and not using family planning was found to be associated with higher odds of anemia among lactating women. This is consistent with evidence from India that showed family planning use was preventive for anemia (AOR = 0.68; 95% CI 0.57–0.80)^31^. Pooled analysis of the 2005 and 2011 EDHS^21^ also showed that family size and non-use of family planning were significantly associated with increased occurrence of anemia. Other studies conducted in Ethiopia has also showed a similar association^13,16,19,32,33^. These are mainly explained with the critical link between family size and the issue of maternal depletion syndrome associated with short interpregnancy intervals. Moreover, it could be linked with food insecurity and nutrient loss due to subsequent pregnancy increasing the burden of the mother. Efforts to maintain an optimal birth interval could reduce the risk of maternal depletion and may allow better food and nutrition security for better nutritional status of mothers^34^. This might be partly explained by the inverse relationship between maternal age and hemoglobin (AOR = 0.8; p = 0.004), which is supported by study done in Southwest Ethiopia (β = − 0.03; 95% CI; 0.04 0.03)^13^.

In contrast, increased maternal age is associated with repeated pregnancy, expanded family size, and increased maternal depletion. However, it could be linked to being primigravida, which is associated with increased blood loss due to pregnancy complications in the earlier periods^2,23^. While evidence from Southwest Ethiopia indicated that maternal age had no any significant association with anemia^35^. While our study indicated that advanced maternal age was associated with a higher burden of anemia. On the other hand, better maternal education reduces the risk of anemia by 75% (AOR = 0.75; 95% CI 0.56–1.03) as compared to illiterates. Those primigravida within the teenager group might have an increased double burden of pregnancy and adolescents. Meeting the iron and vitamin requirements of women during adolescence could contribute to a reduced burden of anemia^11^. Studies have also showed that adolescents were more at risk for anemia (22%)^36,37^, which could be associated with the double nutrient burden. This will allow decision maker to target anemia prevention and control strategies focusing on high-risk groups, with enhanced family planning utilization for achieving optimal fertility level and improve nutritional status^5,14^.

This study showed that women from better economic classes reduced the risk of anemia by half. A study from Myanmar and previous national estimates indicated that better income is associated with a lower risk of anemia which could allow for better food security, educational status, and knowledge of healthy behaviors^3,20,31^. It may also be linked to having better ANC and post-natal visits and adherence to iron and folic acid supplementation^36,37^. In this research majority of lactating women’s having anemia had not history of ANC follow up accounts 509 (49%). This emphasizes the need for enhance nutritional counseling and full package delivery during ANC visit could improve the nutrition intake and health behaviors in post-natal period^14,38–40^.

This evidence clearly shows that anemia is a public health problem among lactating women. Little interventions for addressing anemia among lactating women are there in Ethiopia where routine iron and folic acid supplementations are not in place. Hence, little attention is being given to it despite its adverse consequences, which are mainly related to problems with iron intake^30,41,42^. Intermittent iron supplementation could be an alternative short-term strategy in areas of high anemia burden to supplement existing interventions.

However, the findings of this analysis could be frustrated by many factors. For instance, the old nature of the data could not allow to depict the existing burden of anemia rather it would allow for comparative trends. Hence, the current country insecurity, food security issues and food inflation might have worsened the issue further.

## Conclusions

Anemia among lactating women is a moderate public health problem that needs a targeted and effective intervention to address it. Higher age, larger family size, maternal illiteracy, poor socioeconomic class, and not having family planning use history were found to be associated with anemia. There may be a need for country-specific and cost-effective interventions via enhanced family planning, improving food and nutrition security, and intermittent iron supplementation could be a potential strategy to address the current burden of anemia. Thus, interventional studies and pilots could help to further understand the potential feasibility, compliance and effectiveness of such interventions in addressing anemia among lactating women.

## Data Availability

The datasets used and/or analyzed during the current study available from the corresponding author on reasonable request.

## Abbreviations

ANC: Antenatal care
AOR: Adjusted odds ratio
CI: Confidence interval
COR: Crude odds ratio
DHS: Demographic and Health Survey
EA: Enumeration areas
EDHS: Ethiopia Demographic and Health Survey
PSU: Primary sampling unit
WHO: World Health Organization
